# A Cultural Syndicate of Society and NGOs Challenges To Deter Child Labour Manipulation in Addis Ababa

**DOI:** 10.34763/jmotherandchild.20212504.d-21-00032

**Published:** 2022-04-07

**Authors:** Habtamu Wondimu

**Affiliations:** 1 Sociology Department, Wolkite University, Wolkite Ethiopia

**Keywords:** children, child abuse, sociocultural challenges, self-actualisation, poverty

## Abstract

This study aimed to investigate the existing trends of child labour abuse in Addis Ababa, as well as to examine the sociocultural barriers that impede nongovernmental organizations. This study involves two domestic NGOs (FHIDO and SCFDS) working on child issues. The study used a qualitative approach with 25 participants in in-depth interviews, one focus-group discussion, and six key informants to collect or acquire a full grasp of the issue and to address the study's stated objectives. The finding of this study revealed some psychometric reasons for the children to be engaged in hazardous work activities, including their family background (dynamics), poverty, and self-actualisation arising from contributions to the well-being of the family. Concerning gender-selective norms as a problem, the chosen two organizations are facing a significant task in putting an end to child labour exploitation in the region owing to social gender preference. The cultural and social expectations of men and women make the issue ubiquitous in their communities. Based on the findings of this study, the Winrock International (2008) approach of CIRCLE experience with an awareness-raising plan is recommended.

## Introduction

According to the International Labour Organization (2016), one-fifth of African children have been involved in hazardous child labour tasks, a proportion that is more than twice as high as in any other region. Nine percent of African children are involved in hazardous work, which is the highest rate of any region in the world [11, 33]. Poverty, insufficient welfare provision, poor governance, frequent wars and conflicts, and a lack of societal awareness about child abuse and related problems are the major contributing factors that aggravate abusive labour for children [32].

Agriculture employs 70% of the working population aged 5–15 (34.8%), while industry employs the remaining 30% (21.6 %) [10]. The vast majority of child labour in the sector is unpaid, and children who work in child labour do not have a job contract with a third-party employer, but rather work on family farms and family businesses [6, 10].

Recently, the ILO has used a variety of strategies to prevent widespread abuse of child labour [15]. Although not applicable to some developing countries, the International Labour Organization (ILO) has designed a publicly available and practical strategic database called the Country Dashboard, based on national child labour laws and policies [15]. The ILO [15] argued that the country's dashboard database would begin assessing the application of the ILO's State Enterprise Convention on Children's Rights to Productive Work. Currently, some companies are using this database in their operating systems, and by analysing it, they are formulating assessments of dangerous work activity according to ILO standards [15]. The ILO has also developed a global framework for solving child labour problems. Some state-owned enterprises in some developing and developed countries are obliged to sign these GFAs (Global Framework Agreements), which indicate that the ILO is responsible for child labour and abusive labour obligations. It requires respect and compliance with the labour law norms approved by treaties and laws [17].

Ethiopia is Africa's second-most populous country, with a population of approximately 110 million people in 2019 [4]. Despite impressive economic development, many people in the country remain impoverished [31]. According to Dammert and Galdo [13], 52.9% of Ethiopians are children under the age of 18; among them are 5.5 million orphans and those involved in abusive work experiences. Even though Ethiopia was a founding member of the League of Nations, it is not in compliance with the proclamation strengthening child rights and protection that was recently approved, proclamation number 10/1992 [1, 25]. Articles 4.1, 89.1, 89.2, and 89.3 of Labour Proclamation 1156/2019 (29) state that the average age requirement for employment is 18. Articles 89.3, 89.4, and 186.1 of Labour Proclamation 1156/2019 cover currently banned Occupations for Young Employees [14].

In 2019, the parliament adopted the proclamation of civil society organizations, which replaced a 2009 constitutional provision that restricted the operations of organizations working on child and forced labour challenges in Ethiopia [2, 27]. The 2019 proclamation, which formally repealed the NGOs Charities Proclamation of 2009, dramatically opened up Ethiopia's civic space. Although the government took part in and enforces several anti–child labour programs, these programs would not adequately target sectors with high rates of child labour, such as agriculture and domestic work [27].

As a result, the 2019 proclamation classified nongovernmental organizations and administrative roles into three types [2, 8, 27]: (1) Ethiopian charities and societies which have Ethiopian citizen members and administrators, as well as budgets that are at least 90% locally sourced money; (2) Ethiopian resident charities and societies which have members who live in Ethiopia, but budgets containing more than 10% foreign-sourced money, and (3) Foreign charities and societies formed under foreign laws, employing foreign staff, controlled by foreign nationals, and receiving significant overseas funds.

Among these NGOs, this study included Future Hopes Integrated Development Organization (FHIDO) and Sheger Child and Family Development Charitable Organization, both of which are located in the Addis Ababa city administration's most impoverished sub-cities, Gullele and Addis Ketema, where residents lack the resources and income to live a decent life and the majority of residents live in extreme poverty [5]. As a result, many of their children are engaged in traditional weaving and pottery activities, selling firewood or small goods, and engaging in daily labour.

## Aims

There are various studies conducted concerning child labour impacts and determinant factors across the globe. Related to research conducted by Ahad and colleagues and Jalili Moayad and colleagues [3, 20], which asserted that child labour is highly correlated with stunting, malnutrition, and wasting, Rusavy and colleagues [30] also asserted that the labour could cause delays in children’s genital development and impose shorter stature. Concerning child labour abuse and work-related illness or injuries, the study of Ahad and colleagues [3] shows that there was a significant correlation between the number of working hours by children and the probability of illness or physical injuries. The findings revealed that the children mostly faced backache problems and other health deterioration such as burns and lung diseases. Hosseinverdi and colleagues [19] also reported that child labour is significantly associated with a mental and behavioral disorders, which in turn finally led them to psychosocial difficulties. Despite their positive insights, many child labour–focused studies provide little emphasis on the cultural difficulties of NGOs in addressing children’s issues.

In the areas where this study was focused, most residents (Addis-Ketema and Gullele sub-cities) do not have a regular source of income. For many of them, this means that their children are engaged in several labour activities. The researcher was motivated by his own experience as well as by a request from the directorate of children and women in the Gullele and Arada sub-cities. Children's and women's issues in the study areas confirmed the SCFDS and FHIDO's pivotal roles, but their concern was that the chosen organization had been in the area for over thirty years but had been unable to achieve the desired results. Still, the problem of child labour abuse (CLA) has become prevalent in the two sub-cities. Meanwhile, the question was, while they, of course, have various mechanisms and methods to end CLA, what are the obstacles they face in carrying out their operations, and what are the hidden challenges behind their mission? Furthermore, the woredas’ departments of children and women's affairs and the researcher believe that this research could be a way for us to work together to solve the problem. This study attempted to examine the existing trends of child labour abuse and to identify the societal and cultural challenges that the FHIDO and SCFDS organizations face while working on the issue by taking into account the need for exceptional attention to the problem in the study areas. Furthermore, in the community where this research was conducted, little is known about the difficulty of abusive child labour and its impact on children and the community. Therefore, an examination of NGOs' roles in mitigating child labour abuse and sociocultural triggering reasons or implications was needed to enhance community awareness. The following research questions were raised in this study:

1. What are the current trends in child labour abuse in the study areas? (With this theme, the researcher attempted to answer how self-actualisation, poverty, and the need for family contribution exacerbate the problem of child exploitation.)
2. What are the difficulties that FHIDO and SCFDS NGOs face in addressing the issue of child labour exploitation?

## Material and methods

### Study Design

The study design used was a cross-sectional research design. Since this research aims to examine the sociocultural challenges of selected NGOs and their role in mitigating CLA, the researchers used a cross-sectional design to assess the depth of understanding of children, parents/guardians, and child experts within a specified time [22]. The data was gathered at a single point in time using carefully chosen informants from the target demographics [35]. This study used a qualitative approach to assess the livelihood or lived experience and the existing situations or trends of children who are aged 5–17 and engaged in weaving, pottery organizations, Woyala (assistant drivers), child car-repairers and washers, and so on, which are considered the most exploitive activities [7]. The sociocultural challenges of FHIDO and SCFDS while working on the problem were also investigated using this study design through detailing the lived experiences of the parents/guardians of children and child experts in the chosen organization who know the situation or the problem very well [12, 34].

### Participants, Data Collection Instruments

Purposive (judgmental) sampling was used in this study. In this sampling technique, the researcher decides what he or she wants to learn and then goes out to find key informants who are eager to share information because of their expertise and knowledge [16]. The purposive sampling method used in this research was intended for key informants, in-depth interviews, and focus-group discussions. The primary informants in this research are local community elders or people who have been in the area for more than a year and are well-versed in the issue of child labour abuse. Key informants for this study are responsible personnel who are strongly connected to or work with the selected NGOs. Each of these working children, NGO officers, sub-cities (Kifle Ketema) Woreda administration officers, and officers of the ministry of women and children’s affairs are the key informants. The in-depth interview was particularly designed for people or workers of FHIDO and SCDFC to explore specific methods they utilized to minimize CLA and sociocultural challenges while working on the problem.

In this study, the researcher used the SIMPOC questionnaires developed by ILO (2008). This SIMPOC questionnaire had 126 questions grouped into five parts (PI: Adult survey, PII: household survey, PIII: child survey and interview questions [ages 5–17], PIV: Rapid assessment, and PV: school-based survey). In this case, the researcher selected the qualitative interview questions provided for children found under PIII, and the remaining interview, FGD, and key-informant questions were self-drafted through observation of, and in line with, the societal and cultural sensibilities of the study areas. Accordingly, the number of sample study participants was determined based on the saturation of the information (data) collected, as long as the issues of children's abusive labour tasks and the role and sociocultural challenges that the chosen NGOs faced were concerned. This study purposively selected 8 children, 9 parents/guardians, and 8 NGO participants for the interview. And again, six key informants and one focus-group discussion with eight participants were involved in this study. The interview questions also had a duration of 25 minutes with each student in the form of a one-to-one interview.

An in-depth interview (IDI) was administered to understand the child interviewees' viewpoints about why they are engaged in abusive labour and what kind of labour activity they are engaged in, and to find out the type of support they get from the FHIDO and SCFDS. In this study, the researcher used semi-structured interviews that seemed more like conversations with the informants to understand their views and ideas concerning the problem. The researcher interviewed children involved in abusive labour tasks, NGO personnel working in child departments, and families (community) who are supported by their children. Besides, the participants were asked to reveal their lived experiences of labour engagements. The researcher also contacted staff with expertise concerning children in FHIDO and SCFDS to gain depth of understanding of the challenges they faced while working on the problem, based on their lived experiences in the study areas.

Also, six key informants were included in this study. The key informants that were included in this interview are those local community elders or residents who have lived more than one year in the area and have pretty good knowledge of the issue of CLA. And again, in the key-informant interview, the gender directorates in the sub-cities of Addis Ketema and Gullele, and the NGO personnel in the selected organization, are also included. For this purpose, interview guides are prepared that elicit responses on various aspects related to the specific objectives of the study under investigation.

Another instrument that the researcher used to collect data from the target population was focus-group discussion (FGD). The researcher attempted to conduct one focus-group discussion that comprised eight discussants. The selection of discussants for FGD was made through purposive sampling; the researcher selected discussants who were willing to provide information stemming from their experience and knowledge. The researcher organizing the FGD included those parents or communities who get support and means of subsistence from their young children as the prior inclusion criteria. The second inclusion criteria was that residents should have lived for at least 12 months in Addis Ketema, Kifle Ketema, and Gullele, where FHIDO and SCDFS are work, which means people who reside in these areas for less than 12 months were excluded. People who have no children, or have no experience of the problem, and or who have mental issues/disorders (identified by medical checkups and with the help of the institution's nurses) are not to be included in the FGD.

Due to the hideous corona pandemic, the researcher was not able to conduct focus group discussions with the target groups. However, to achieve the intended quality of data and level of saturation, FGD seems to the researcher to be necessary. The FGD was held through WHO prevention mechanisms or requirements. Participants were enjoined to keep their distance, wear masks, and use sanitizers. The researcher also used nonparticipants' observation of the problem. This was carried out through a personal transect walk in the field areas. The nonparticipant observational method magnifies the existing trends of serious child labour abuse without any biases or prejudices about the working conditions and related issues/hazards while they are at work with employers, who may be their relatives or families. The researcher observed how they are engaged in abusive work activities (pottery, Woyala, selling firewood, selling small goods, and so on) and the NGO welfare services provided for children 5–17 years old.

### Data collection procedures

Data collection began with a formal letter of request written by the Sociology Department at the University of Gondar to the FHIDO and SCFDS. After gaining approval, eligible participants were identified based on the study's inclusion and exclusion criteria and asked if they would be willing to provide information about the issue under investigation. Before that, the researcher gave a brief explanation of the study's purpose and the issue of confidentiality. Before getting involved in data collection, the researcher provided orientation to assistant data collectors on how to handle the required interviews, and how to manage focus-group discussions. The respondents, especially the children, were informed that they could disagree to participate without their willingness to reveal the intended information. We also asked of children and parents/guardians permission to include them in the paper; the children and parents who were not willing had the right to not be involved. The researcher gave them each an informed consent form, read it aloud to the informants, and answered their questions about their agreement and other confidentiality issues. After hearing and reading about informed consent, the adult participants or parents/guardians agreed to participate in the informed consent for themselves and their children.

### Data Quality Assurance

To assure the validity of the data, a pilot study was used to improve the quality of the questions; formats, arrangements, and language were used to enhance the validity of the data. The questionnaire was translated from the English version into Amharic, and it was retrieved back to ensure the relevance of its information. The arrangement of the survey questions was checked, along with the instruments. Also, the researcher assured the validity of data using distinctive technical means. The researcher promoted the quality of the study through formulating a good rapport. Clarifying the objectives of the research to respondents, approaching respondents in friendly ways, and getting trust and respecting the cultural values of the participants were some procedures to improve the trustworthiness of the information. Furthermore, data triangulation is often helpful: we used several techniques for the same method so that one technique's weakness is offset by the other's strength [21]. To address the credibility of the study data triangulation, references to articles from peer-reviewed Scopus/WOS journals were used [7].

### Data Analysis

The qualitative data collection instruments, such as in-depth interviews, focus-group discussions, and nonparticipant observations, aid the researcher in developing the thematic analytical strategy for this study [24]. The data collected from in-depth and key-informant interviews and FGD were analysed thematically or qualitatively to explore the existing trends of CLA in the study areas and analyse the sociocultural challenges that the selected NGOs face while working on children's problems. Accordingly, the data collected from the study participants were collected using the tape recorder and by taking notes, respectively. However, the data was reviewed through repeatedly reading and listening to the recorded data using a tape-recorder as data collection equipment. Finally, the participant reflects on what they have on their minds using Amharic (the local language), and the researcher transcribes and translates the data from Amharic to English without resigning its content effectively. And then, the generated data was organized through paragraph code or through the use of respective themes that go along with the specific objectives stated to be examined in this study. Themes and sub-themes were used to create a meaningful conclusion that was also coherent and understandable. The study analysed the data into two themes: (1) An examination of current trends in child labour exploitation was held through the three theories (self-actualisations, family contributions, and poverty) that indicate the reason that the children in Addis Abeba's Gullele and Addis-Ketema sub-cities engaged in the abusive workforce. (2) Challenges of the chosen NGOs (FHIDO and SCFDS) were assessed through labeling gender-selective norms and social perception toward child labour and NGOs as a challenge.

Accordingly, to gain a detailed examination of the personal lived experiences of the participants, interpretive phenomenological analysis was used. This is because to understand the depth of experience of children in their prevailing or engaging areas, to make sense of their own experience of child expertise, and to inform parents/guardians of children about the involvement and challenges of FHIDO and SCFDS, a thorough understanding of the personal history of the participants is essential.

## Results

The study's findings were examined in light of the specific objectives, and then analysis and interpretation were performed using triangulation, with certain qualitative topics examined independently. This study included 25 participants in in-depth interviews, one focus-group discussion, and six key informants to obtain or gain a thorough understanding of the problem and to address the study's stated objectives.

### The existing trends of child labour abuse

#### Self-Actualisation

During the interview, an anonymous participant in the study confirmed that engaging in various types of labour activities makes them quite independent and mature. In the interview, a 17-year-old female child informant confirmed where she worked and how the work she did helped her mature:

*I usually work on household chores, but I used to sell chewing gum and soft paper on the side. I'm not sure how much money I can make because I get paid as things are sold. Most of the time, I receive between 30 and 60 Ethiopian birrs, and most people give us money if they believe the situation is dire, but they are in a good mood or condition. They asked for assistance, were sad, and gave us some extra money. Even though the money I get is small, it makes me feel I am mature enough to do things in a better way. Because the money I receive enables me to meet all of my needs as well as those of my family*.

Another 15-yearold child participant in the in-depth interview told me that:

*I feel I have become mature by the thing I have done right now. I am working in the two places. The first place is Garaj (car repairing), and the second is on the car washing places when there is no car to be repaired in the Garaj. I feel mature because, before I engaged in these working activities, I always ask my parents to buy me my needs, and I was dependent. But after having this job at least I began to be aware of the value of myself and what should I have to do next. Due to the work activities engaged now, I can plan what should and what will happen and in what ways should I handle all my problems. I used this money to support the family partially, and I use the remaining money to afford everything that I require*.

17-year-old female in-depth interview participant also argued how the work she performed made her more mature and multidimensional than any of other her friends, and how she carried on her daily labour tasks besides formal education.

*I work in the pottery organization, and I’m being assistant of the others which means when you are a fresh you take the position of assistant of the main employees who are working Jebena, Ensera, Dest, etc. Of course, the work that I have done makes me think I am capable enough for myself. You know when you are a female you face so many natural challenges like (there is menstruation period, bullying from males, embarrassment for our femininity, etc.). However, this work enables me at least to show how I can sustain myself without the support of anyone and to handle these challenges*.

Thus, the data shows that most of the sample children, even though they received little money, were satisfied by the things they were doing because it made them mature and dependent on themselves. The psychological independence allows them to sense or ignore the need for further assistance, leading them to the conclusion that they can earn their own pocket money without the help of their parents. Some psychometric reasons make children engage in the abusive labour force. Besides, of course, some light tasks help children mature and boost their ability to engage with various issues. This study for psychometric reasons found that children in the study areas were actively engaged in hazardous work scenarios because they feared family breakdown and divorce as a result of economic insecurity. Children sometimes were involved in CLA in order to prevent confusion among parents and to recover from deprivation. In addition, the study found that some children surveyed had loneliness and hopelessness due to the emotional negligence of their families. Family affection and lack of affection are both associated with child abuse.

#### Family Contribution and Poverty

In the interview, the study's child participants revealed that the main reason they engaged in the abusive labour task was to help their families get out of poverty. For example, a 14-year-old assistant driver (*Woyala* in the local language) confirmed how he was involved and performed his labour tasks related to his education,

*It's been 2 months since I started at this job. I come from a poor household and, like many others, could neither afford my school costs nor necessities such as clothes, books, uniforms, and so on. I worked as Woyala by round. In other words, I am the night round up to 10 p.m., and there is an employee who works the whole day in the same car. As a result of my education, I usually carry on my job in the evening or at night after 6 p.m. and up until when the work is completed, but we usually return home at 10:30 p.m. because our car is a mini-bus which carries 15 people and there are Lada or taxis who work the entire night, and we cede our place to them after 10:00 p.m*.

Another garage worker in the interview said,

*I started working at Garaj this year. I was a grade 9 student and ceased my studies due to the quarrel I created with my teachers. The school principals immediately punished me for not attending my education for one year, which means I can start my education in the next year. However, I am a full-time worker now. When I was in school, I performed my labour tasks before and after going to school, but much of the work was done between Saturday and Sunday. My family is among those in society who can't afford or fulfill their basic needs, and we are in a hideous situation. As a result, most of our family members engaged in various paid-labour activities. During the school season, I often miss my classes to support my family in the household, and when I do, I suffer from an injury or illness due to my work situation. I hope everything will be going to be better*.

In the same vein, another 17-year-old child participant (assistant driver) in the interview stated that he was involved in labour tasks to support his family, but this, unfortunately, caused him to be weak in his education. The reflection is as follows:

*I am a 10th-grade student and, at the same time, working as an assistant driver, or what we call "Woyala". It has been two years since I started working at this job. Like many other people, I am from a poor family and can't afford my school expenses and my basic needs, like clothes, books, uniforms, and so on. I am not satisfied with my work, not because it is so difficult, but because it can hamper my schooling. I was a top 10 scorer before I joined these activities, but now I have started to miss my classes to work to support my family. This task may damage your throat and make you miss your education. As a result, I became very weak in my studies. Even if sometimes the teacher asks me "are you in this class", I don't blame them because, as I told you, due to this job, I missed so many classes and I go to school when I get a convenient time*.

Similarly, key informants from the chosen NGOs make similar confessions:

*Most families, as you see, do not have enough income to afford the basic needs of their children, such as food, clothing, shelter, medication, and school-related costs. On the other hand, they lack proper knowledge of how to generate income through different ways, like increasing income through micro-enterprise; practicing different types and mixed businesses; undertaking off-time activities to create additional income and income generation schemes. So, developmental consequences of missing educational opportunities as well as the mental and emotional harm of being forced to work long hours*.

In a similar vein, one FGD participant described how her daughter was employed and supported by the chosen NGOs:

*I worked in pottery for more than twenty years and my daughter started working here when she was 12, and she is now 16. We joined the "Ensera" pottery factory three years ago, located in Kechene, northern Addis Ababa. As a result of my daughter, I am supported by SCFDS, or we call CCF common sense. The organization of SCFDS told us they will be involved in regulating our children's modes of payment and working situations through outlining proclamations and regulations and collaborating with federal government child rights conventions*.

Thus, as a result of assisting family business and household chores, the majority of respondents were missing their education and engaged in the abusive labour force. Many children work or participate in CLA because their families require help in providing for their families and contributing to unpaid household labour. Because children frequently see themselves as members of the family unit, the majority of children believe that helping around the house is possible, especially if family survival is at stake.

## Observational results through autographs

As the researcher saw the homes of the working children during the fieldwork, the way the children were living was heartbreaking, and one might argue that the youngsters should work to alleviate life in the family to some degree. In other words, labour is required for these youngsters to meet their family's fundamental necessities, especially food. The increasing cost of living is one of the factors contributing to the economic insecurity of working-class parents and impoverished families. Food costs have risen, putting good nutrition for many families out of reach. This issue, either directly or indirectly, compelled youngsters to participate in productive activities to get something valuable in cash or in-kind for themselves and their family’s survival.

**Figure 1:**
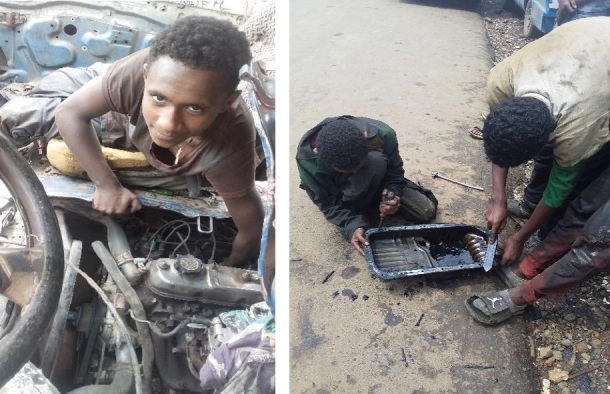
14- and 16-year-old children working in the garage

**Figure 2:**
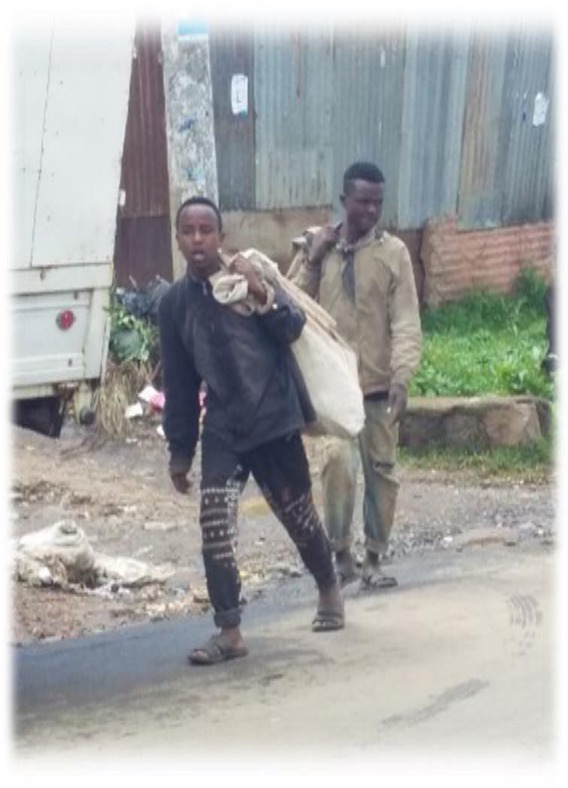
Children16 and 17 years old in the street vending

**Figure 3:**
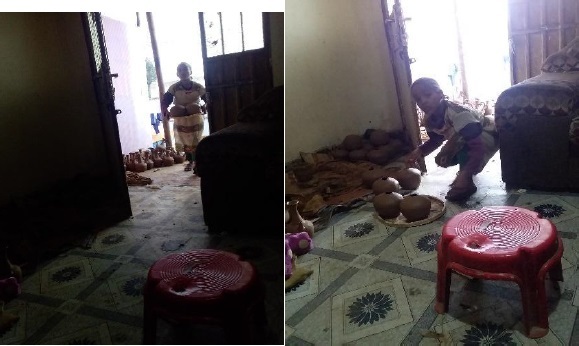
Nine-year-old child working pottery at home

**Figure 4:**
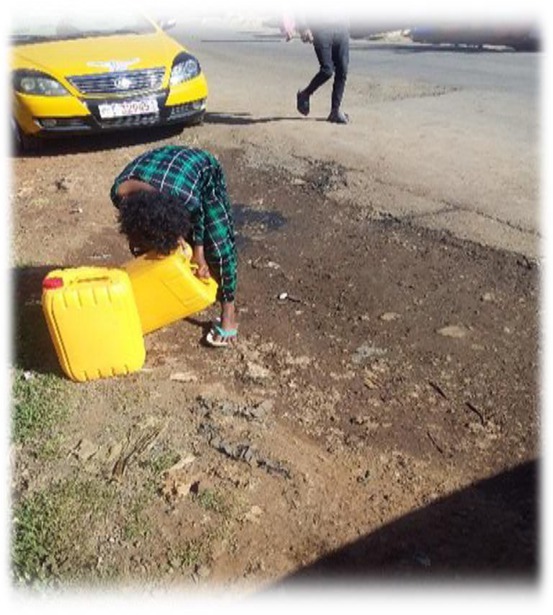
Ten-year-old child carrying out domestic chores

**Figure 5:**
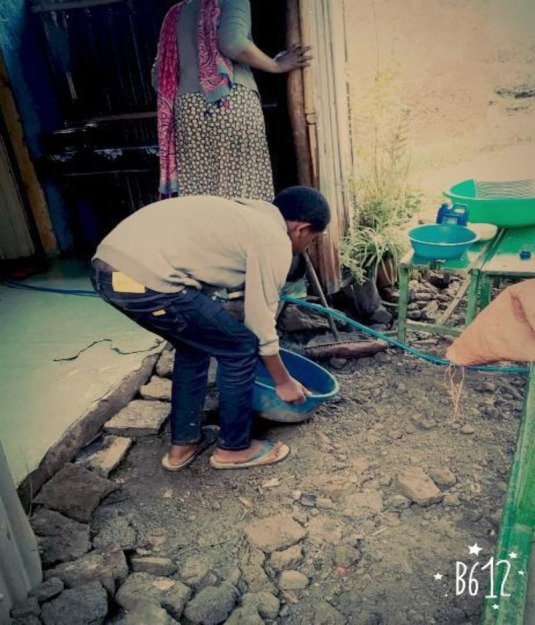
14-year-old child supporting his family at home

**Figure 6:**
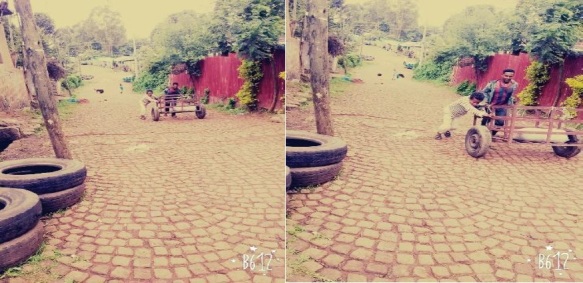
A 12-year-old child selling charcoal using a simple cart

**Figure 7:**
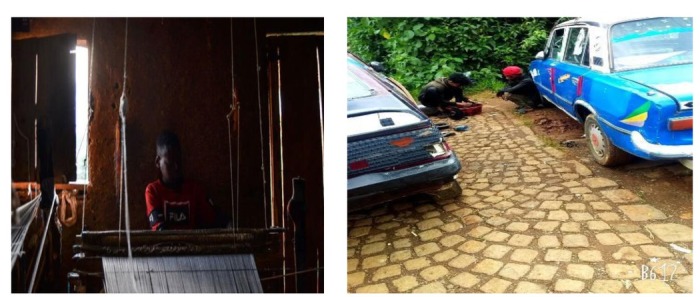
Children working on weaving and garage

The data in Figures 1 to -8 shows children who are engaged in domestic home duties such as fetching water and cleaning the house, and in the public sphere, the children who are working as assistant drivers (Woyala), and car repair. Also, the researcher noticed that the children also worked at the pottery work at the house as well as the Ensera pottery organization. Finally, the researcher observed the children working at the charcoal-supply job utilizing a basic cart.

**Figure SEQ Figure \* ARABIC 8:**
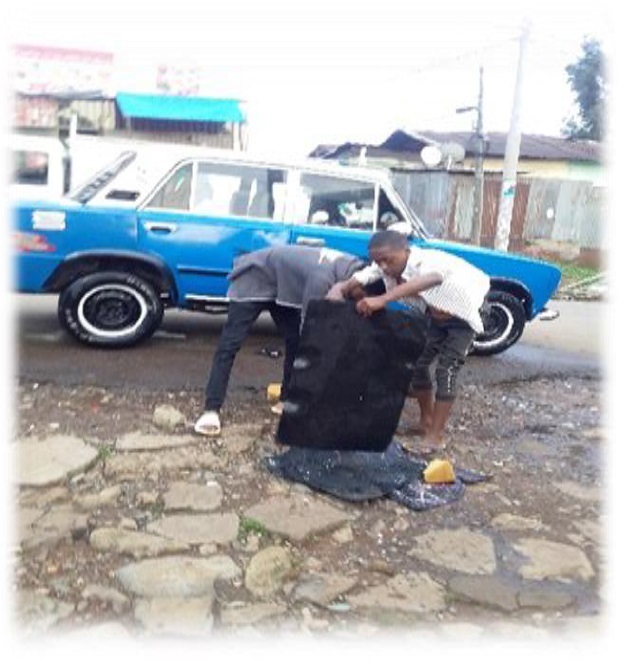
A 14-year-old child engaged in car washing activities

According to the findings of the fieldwork, child labour must be prioritized. In general, a significant percentage of youngsters work in the fields of pottery, household duties, and the garage. In the studied regions, the issue is much too severe. Despite the researcher's preference to concentrate on working children, the majority of youngsters in Gullele and Addis Ketema sub-cities work: carrying commodities, street vending, gathering and selling fuelwood, shoe shining, taxi calls, car washing, working as assistant mechanics in tiny garages, and so forth.

The researcher's observations confirmed this reality about the family environment of child workers. As a result, it is reasonable to conclude that children of impoverished parents are more likely to enter the labour force than children of contented parents. Furthermore, many parents of child labourers were engaged in other economic activities such as gathering firewood, preparing food for sale, and everyday labour to support their families. Worse, they do not get enough money from such jobs to meet at least their children's basic requirements. In terms of the educational backgrounds of the parents of the working children in the study area, the majority of them were illiterates, but they supported their children's education. The hired and self-employed child labourers were living in deplorable conditions, unable to meet their basic needs. Many of the children were unable to eat at least twice a day.

The researcher observed a four-meter-wide-by-nine-meter-long room in Gulele sub-city Woreda 07, near Kechene, that functioned as a place to live and work for about 37 family members, including child labourers. Another issue I saw among the Kechene child labourers was a lack of water and light. Working children who live far away from their parents live in desperation. Aside from their terrible living conditions, the kind of food they ate was another indication of the young labourers' miserable lives. Child labourers, particularly child weavers, take leftovers, loaves of bread, or bullei (local language) from several local eateries to satisfy their dietary needs.

## Challenges of FHIDO and SCFDS in mitigating CLA

### Gender-selective norms as a challenge

The study of child experts in-depth interviews, key informants, and one FGD (8 participants) revealed that gender-selective norms that society entertains has an enormous impact on children engaged in abusive labour tasks. For example, one participant from FHIDO claimed,

*Ooh…Gender-selective norms and society’s male-oriented view have a significant impact on working in our organization, although relatively little in the case of CLA. Because the culture in which our organization operates divides fundamental occupations between men and women. And they expect ladies to undertake all the home or domestic duties, while men are supposed to go shopping and gather firewood, although this is no longer the case. Everything is done on a stove or with intensive technology, and the majority of the hard and repulsive labour is done by women. The majority of men are in school studying or are encouraged to actively participate in their school*. (in-depth interview)

Another participant from SCFDS, a child expert, supported that:

*We all know what kind of society we have. Society favors women rather than males in all household chores or work. However, many females in our community suffer from CLA due to our bad rituals, practices, and norms. This should be solved unless we can't bring or mainstream the gender gap*. (in-depth interview)

In this section, the researcher has used the community site program coordinator as a key informant. Despite the aforementioned interviewees, this team leader argued that gender-selective norms are not the major challenge to the SCFDS organization and stated,

*According to the evidence we have, society's gender selection norm does not have a negative impact on CLA levels. My minor worry is that society discourages girls and does not enable them to pursue formal education. Most females are expected to perform all the home labour, and society encourages women to marry earlier and take on all duties because they think it will help them mature*.

And another participant actively working in the department of child education, an officer in FHIDO, tried to make a clear argument through integrating a patriarchal society. The participant said,

*We are all aware that the patriarchal system is practiced in the majority of Ethiopian communities. That refers to the male-dominated family that we all have. In our society, however, there has lately been a little favoritism toward men, in contrast to the previous system. Females bear enormous responsibilities, which impact their self-reliance, cognitive capability, and sense of dependence in every way. Of course, there is a gender bias in our area, where females are not encouraged to fully participate in all aspects, which causes them to be overly precise in abusive domestic chores because society is unwilling to educate their daughters on how to negotiate with abusive work tasks and to understand their responsibilities and rights*. (in-depth interview)

The study results regarding gender-selective norms as a challenge, therefore, revealed that the selected two organizations are now being highly challenged to bring an end to child labour abuse in the area due to societal gender favoritism. The cultural and societal expectations of males and females, however, make the problem rampant and prevalent in their residing areas. Even though they have worked through the outlining of various strategic measures, the participants from FHIDO and SCFDS claimed that these abusive cultural norms mean that the organizations the do not work to mitigate the gender gap in engagement in abusive child labour tasks.

### Societal perception toward NGOs as a challenge

The result of this study asserted this limited perception as a major difficulty for NGOs working with the community openly. For example, the first child officer participant in FHIDO and

SCFDS argued,

*Because the societal perspective or attitude in CLA is extremely limited or little, changing their attitude requires a lot of work, causing the organization to lack and fail to successfully integrate with them. We, as well as the government, need to put a lot more effort into this if we don't want the issue to spread throughout the area. We are focusing heavily on community attitudinal transformation in collaboration with the government*. (In-depth interview)

Despite the facts detailed by the previous interviewees, another participant from FHIDO claimed:

*I believe that societal perception does not help to reduce the severity of the FHIDO problem. Because the organization is also working to alleviate the issue by collaborating with other funding agencies. The main goal is for children to have a safe environment and a better way of life, and our organization works patiently to achieve this goal. The most difficult aspect of the organization is the societal loss of enthusiasm in CLA or their deteriorated livelihood. Furthermore, we also believe the societal negative attitude and the government's lack of attention to CLA exacerbate the issue and force the organization to perform poorly. Finally, the organization does not receive a sufficient response from the government, or funds*. (in-depth interview)

Another key-informant child and women or gender directorate from Woreda 07, claimed that parents/guardians involved in the community consultations reflected that they never go to school to follow up on their children's progress unless they are called. Whenever children drop school and parents are called to the school administration, they report that they are unable to influence them to attend education. This lack of belongingness, commitment, and interest on the part of parents in children’s education mainly stems from their low level of awareness of the importance of education and their poverty level. The major problems identified as facing children in their low education performance are as follows: (1) Low awareness of family/guardian of children's education, absence of follow up, unwillingness to send children to school for club participation and library reading; family workload on children; (2) Unfriendly school environment, playgrounds (dusty, rough, scarce play materials, absence of recreational site), unfulfilled gender club, no water access for toilets, fence, uncomfortable laboratory, and pedagogical center, (3) students’ poverty (family unable to cover basic needs of clothing and food, school materials for their children); and (4) high dropout and repetition rate in schools and presence of out-of-school children in the target operational area (key-informant interview).

The participant in the FGD said that the reluctance from society is not the major problem; rather, the misunderstanding between the society and the organization aggravate the problem immensely. Their argument is as follows:

*Of course, most of the NGOs faced various challenges from the community. We are being supported by the SCFDS recently. However, there is a misunderstanding between the community and the organization. For example, some programs engage childhood illness and malnutrition, however, their project on this issue was unable to ge tto its goal because the society doesn’t want the training; rather, they have to support and show us financially the ways that poor personal and environmental hygiene practice and lack of quality of education do damage. Also, we want them to show us how we can create a safe and caring environment. We currently want a leader instead of managers, they should lead us in addressing child labour abuse through providing various financial instruments. Therefore, the challenges emanated from the organization's unwillingness to address the problem at the bottom. So, failing to bring the desired outcomes in terms of CLA resulted from the misunderstanding of the organization what the society needs concerning the value and norms of the society*.

As a result, the study in this section implied that societal perceptions of child labour abuse and the role of NGOs are limited. However, due to a lack of societal awareness, these selected NGOs lost millions of dollars. Although organizations provide educational vocational training all year, there is a lack of expertise to educate the community on how the training is implemented in the real world.

## Discussion and Conclusion

The goal of this research was to look into the current trends of child labour abuse in the Addis Ketema and Gullele sub-cities, as well as to address the sociocultural barriers that impede the organization's intentions. The study involves two domestic NGOs and children supported by these organizations, FHIDO and SCFDS. To gain a thorough understanding of the subject under investigation, the researcher employs a qualitative data collection approach (FGD, in-depth interviews, key informants interviews, and nonparticipant observations). The research's main findings are thus discussed in this chapter following its specific goal and theoretical framework.

### The existing trends of child labour abuse in the study areas

To investigate the existing trends of child labour abuse in the research area, qualitative (thematic analysis via in-depth and key informant interviews and focus groups, observation) approaches were used. It was discovered that the children in the study were involved in hazardous work activities as a result of their family background (dynamics), poverty, and self-actualisation to contribute to the well-being of the family. The result has also shown that salaries of children from their jobs is low but that children were satisfied by the things they are doing because it makes them mature and independent. These research findings are somewhat similar to those of Heissler and Porter [18]. They discovered that exceptions are influenced by household characteristics, but are primarily driven by intergenerational interdependence. Furthermore, such exceptional cases are not coincidental; a child's work is affected by poverty and changing family circumstances. In the same vein, the findings are remarkably comparable to Rowan’s [29] conceerning self-actualisation. Rowan [29] dictates that dependence can be psychological or economic. In terms of financial independence, children want to find ways to escape their family's poverty. As a result, people in some industries are forced to accept exploitative labour. Because of their psychological independence, they can detect or ignore the need for additional assistance, leading them to believe that they can earn their own pocket money without their parents' assistance [26, 28]. According to research on children's contributions to the household economy in Ethiopia, participating in paid job activities gives children pride in themselves and helps to build children's sense of their human and psychological abilities [18].

Many youngsters work or participate in CLA because their families require assistance; these children aid in supporting their families and contribute to unpaid domestic labour. Because children frequently perceive themselves as members of the family unit, the majority of children feel that helping around the house is possible, especially if family survival is at risk. This result is also similar to the findings of Bourdillon as well as Letsie and colleagues and Özaslan [9, 23, 26]. This indicates that, as a result of assisting family business and household chores, the majority of respondents were missing their education and engaged in the abusive labour force.

### Challenges of NGOs in mitigating CLA

The researcher has compiled a list of the challenges that the selected NGOs faced while combating child labour abuse, which is primarily related to societal values and norms. The section was thematically analysed, and the researcher contacted some selected child experts who are actively working in FHIDO and SCFDS, as well as key informants from the organizations' community site program coordinators and the Woreda gender directorate, as well as one focus-group discussion with eight participants.

As a result of the study's findings addressing gender-selective norms as a problem, the chosen two organizations are now facing a significant task in putting an end to child labour exploitation in the region owing to social gender preference. The cultural and social expectations of men and women, on the other hand, make the issue ubiquitous in their communities. Despite having worked through the development of different strategic solutions, participants from FHIDO and SCFDS stated that these cultural norms force organizations to disregard or downplay the gender disparity in the participation in abusive child labour activities. The study on social views of NGOs and CLA as a difficulty indicated that public perceptions of child labour exploitation and the role of NGOs are very limited. However, due to a lack of public awareness, these chosen NGOs lost millions of dollars. Even though organizations offer educational vocational training all year, there is a shortage of competence to educate the community on how the training is applied in the actual world. According to the respondents, another difficult factor that encourages and escalates the issue of CLA is the community's reluctance or lack of interest in working with NGOs owing to a negative attitude. This study also indicated that the problem came not just from society's perceived cultural background, but also from the community's or parents'/guardians' lack of interest, and lack of confidence in working with NGOs. In addition, religious misconceptions have a significant impact on CLA supplementation. The interviewees, on the other hand, said that language barriers had little or no impact on the CLA. Although the majority of people in the region have differing origins, they speak the same language and communicate with the organization in Amharic, so the impact is limited. In the same vein, the findings indicated that cultural values related to child development, gender, marriage, religion, and ethnicity may affect children's labour in low-income countries. This could be a barrier for humanitarian workers or NGOs involved in adequately mitigating and addressing the problem. In many cultures, children are expected to follow in their parents' footsteps and learn the trade from an early age. Some cultural beliefs may lead to the mistaken belief that a girl's education is less important than a boy's education, and as a result, girls are forced to work as domestic service providers as children. These gender biases and negative societal norms and beliefs undermine and exacerbate the cross-cultural ethics of children's NGOs in addressing the issue.

## Study Limitations and Recommendations

This study's findings are subject to some limitations. The child participants were unwilling to describe inflictions or hurts related to the activities they participated in, no matter how heinous they were. Because they are unwilling to express their pain explicitly, this study lacks external validity. As a result, the extent of external validity remains unknown, necessitating additional mixed research involving both qualitative and quantitative approaches. The quantitative study allows for the inclusion of the opinions of several sample children in the study areas to determine the number of hours they work per day, weekly, and monthly, and compare them to the federal working hours limits outlined in conventions and proclamations. Second, comprehensive data and studies were scarce in the study area. Finally, the researcher employed a cross-sectional study design. However, the prevalence rate of child labour abuse and cause and effect relationships were difficult to determine using this study design. A replication of this study with qualitative and quantitative data and significantly larger sample size may thus be extremely beneficial. Furthermore, the findings of this study concerning child labourers have implications for policymakers working with NGOs. The government must help child labourers and their families improve their living conditions by improving the quality of their educational and employment opportunities.

To address the sociocultural difficulties of NGOs in reducing child labour exploitation, the Winrock International (2008) approach of CIRCLE experience with an awareness-raising plan is recommended. The primary target audience of the strategy was impacted or at-risk families, including children. A substantial number of initiatives, however, are targeted at reaching state actors at the central and local levels, especially education and social welfare agencies, as well as local employers. Faith-based and religious organizations, on the other hand, are encouraged to engage in child labour prevention and may be successful in promoting good change. I think this strategy is all-inclusive and compatible with the community, like the areas this research included. Many poor families look at education in terms of how it will support the family in the long term. If children can complete their education cycle and emerge with a set of skills that can facilitate their access to decent work with improved working conditions, this will make the program more appealing to parents, who are the main family decision-makers. However, the organizations or the chosen NGO’s training should include acknowledging that technical skills, while required for employment, are not sufficient in themselves. This research also suggested that the issue stemmed not only from society's perceived cultural context but also from the community's or parents'/guardians' lack of interest and trust in working with organizations or NGOs. However, the chosen organizations should redouble their work to increase community trust in the organization through regular community consultation and feedback sessions.
